# Ciliome resequencing: A lifeline for molecular diagnosis in LCA

**DOI:** 10.1186/2046-2530-4-S1-P55

**Published:** 2015-07-13

**Authors:** I Perrault, S Hanein, M Nicouleau, S Saunier, C Bole, P Nitschké, O Xerri, N Delphin, A Munnich, J Kaplan, JM Rozet

**Affiliations:** 1 Genetics in Ophthalmology, U1163, Hôpital Necker, Paris, France; 2 Department of Genetics, Hôpital Necker, Paris, France; 3 INSERM UMR 1163, Molecular bases of Hereditary Kidney Diseases, Paris, France; 4 Genomics Plateform, Hôpital Necker, Paris, France; 5 Bioinformatics Plateform, Hôpital Necker, Paris, France

## Aims

Leber congenital amaurosis (LCA) is the earliest and most severe retinal dystrophy. It occurs as non-syndromic or syndromic. 26/37 LCA genes are important to ciliary function and account for < 1/3 of cases. These cases develop- or are at risk to develop- skeletal, renal and/or neurologic symptoms. Here, we assessed efficiency of ciliome resequencing (CR) as a tool for molecular diagnosis and patient care.

## Patients and methods

The DNA of 60 unrelated young children with LCA was screened for mutations using a custom 5.3 Mb Agilent SureSelect Target Enrichment library which captures 32,146 exons of 1,666 genes selected form cilia databases. Segregation analysis of rare candidate variants was performed by Sanger sequencing.

## Results

Biallelic disease-causing mutations in known genes were identified in 17/60 patients (30 %): *CEP290 *(n = 4), *CRB1 *(n = 4), *RPGRIP1 *(n = 2), *LCA5 *(n = 1), *IQCB1 *(n = 2), *IFT140 *(n = 2), *AHI1 *(n = 1), *ALMS1 *(n = 1). In addition, 3/60 patients harbored biallelic mutations in three novel genes which screening in additional non syndromic and syndromic LCA cases allowed identifying additional mutations in 2/3 of them.

## **Conclusions**

The identification of mutations in known and novel genes in 33 % of the cases, makes targeted sequencing an interesting alternative to exome resequencing. The identification of mutations in several genes responsible for syndromic LCA in young children with no overt extraocular expression demonstrates the importance of NGS-based molecular diagnosis to set-up a rational and efficient follow-up of patients.

